# Editorial: The Brassicaceae—Agri-Horticultural and Environmental Perspectives

**DOI:** 10.3389/fpls.2018.01141

**Published:** 2018-08-27

**Authors:** Naser A. Anjum, Sarvajeet S. Gill, Om P. Dhankher, Juan F. Jimenez, Narendra Tuteja

**Affiliations:** ^1^CESAM-Centre for Environmental and Marine Studies, University of Aveiro, Aveiro, Portugal; ^2^Stress Physiology and Molecular Biology Lab, Centre for Biotechnology, MD University, Rohtak, India; ^3^Stockbridge School of Agriculture, University of Massachusetts Amherst, Amherst, MA, United States; ^4^Institute for Scientific and Technological Research, San Luis Potosí, Mexico; ^5^Plant Molecular Biology Group, International Center for Genetic Engineering and Biotechnology, New Delhi, India

**Keywords:** brassicaceae family, agricultural-horticultural perspective, environmental health, plant breeding, crop improvement

Human health is closely related with the environmental health; therefore, efforts are being made to improve their interrelationship. In this context, a number of plant genera have been identified which can significantly contribute to the protection of the environment as well as human health. Notably, Brassicaceae is one of the prominent plant families with *Arabidopsis, Alyssum*, and *Brassica* as model plants; *Boechera, Brassica*, and *Cardamine* as developing model generic systems; and radish, rocket, watercress, wasabi, horseradish, vegetable, and oil crops as cultivated plant species. Thus, most members of Brassicaceae have been the subjects of exhaustive research mainly due to their innumerable multidimensional roles in the current context. The Frontiers Research Topic “*The Brassicaceae—Agri-Horticultural and Environmental Perspectives*” aims to highlight major current global research outcomes in this direction. This research topic incorporated 28 publications including 27 research papers and one review article. Molecular-genetic insights into agri-horticultural aspects were dealt with in 20 of the publications, six reports were on the stress management and nutrition aspects and two reports on environmental perspectives.

## Yield and nutritional quality

Improving yield and nutritional quality has been an important focus of the agri-horticultural studies on the Brassicaceae family members. The formations of the flower and siliques directly affect seed yield (SY); hence they are considered important for *B. napus* production, where delayed planting can impact SY. Additionally, the period from floral meristem differentiation to budding governs effective flower and siliques formation (Zhang et al.). In this context, maximizing effective flower numbers prior to budding and reducing the degradation of the floral meristem can improve silique numbers and its final formation, respectively. *Brassica* oilseed genotypes with apetalous flowers are a component of the high-yielding ideotype and also of particular interest in breeding programs. *B. napus* possess nine consensus quantitative trait loci (QTLs) present on the A3, A5, A6, A9, and C8 chromosomes (Wang et al.). Notably, the population with QTLs *qPD.C8-2* and *qPD.C8-3* can exhibit a high heritability of petalous degree (PDgr) and less sensitivity to environment. In another report, among seven major QTLs identified by Zhao et al., three and two QTLs were for SY and seed weight (SW), respectively; whereas, one QTL each was for branch height (BH) and branch number (FBN). Particularly, the expression of QTLs for SY can occur stably in winter (*cqSY-C6-2* and *cqSY-C6-3*) or in spring within the rapeseed cultivation area (*cqSY-A2-2*). Seed yield losses due to shatter have been a great issue in commercial harvesting of *Brassica* genotypes. In particular, *B. napus* exhibits a multigenic inheritance for pod shatter resistance. In a diversity panel, doubled haploid and intermated possess six QTLs for resistance to pod shatter (Liu et al.). These QTLs are located on chromosomes A01, A06, A07, A09, C02, and C05, where QTL *qSRI.A09* and *qSRI.A06* can occur across environments.

In radish (*Rhaphanus sativus*), information is scanty on the occurrence of cytoplasmic male sterility (CMS; a maternally inherited trait incapable of producing functional pollen) at the posttranscriptional level. *R. sativus* male sterile line “WA” and its maintainer line “WB” were revealed to involve a potential miRNA-mediated regulatory network of CMS during anther development (Zhang et al.). In *R. sativus*, premature bolting results in poor root growth and reduced harvest. Differentially expressed genes (DEGs) related to *R. sativus* bolting and flowering involve the model of 24 miRNA–DEG pairs (Nie et al.). Particularly, the intricate genetic networks of bolting and flowering in *R. sativus* involve the pairs including miR5227–VRN1, miR6273–PRP39, and miR860–NF-YB3. Notably, the major molecular mechanism underlying the complex *R. sativus* taproot development process is in its infancy. *De novo* taproot transcriptome sequencing and analysis of major genes involved in sucrose metabolism discovered a total of 103 unigenes, encoding eight enzymes involved in the sucrose metabolism-related pathways (Yu et al.).

It is possible to modify the content of secondary metabolites, including glucosinolates (GSLs), and obtain vegetables enriched in these compounds, which are related to plant defense and human health. The developed six *Brassica oleracea* var. *acephala* genotypes exhibit high and low contents of three major GSLs namely sinigrin (SIN), glucoiberin (GIB), and glucobrassicin (GBS) (Sotelo et al.). Compared with the divergent selection, the use of mass selection can be an efficient way of modifying the SIN and GIB concentrations that are related with the GSL–ALK locus that, in turn, varies with the *CYP81F2* gene expression. Storing the whole heads of broccoli (*B. oleracea* var. italica) at 20°C for 24 h exhibits several specific phenolic compounds such as 1,2,2-trisinapoylgentiobiose (2,2-TSG), 3-O-Caffeoylquinic acids (3-O-CQA), 1,2-disinapoylgentiobiose (1,2-DSG), 1,2-diferulolylgentiobiose (1,2-DFG) and 1,2-disinapoyl-2-ferulolylgentiobiose (1,2-DS-2-FG) (Villarreal-García et al.). Additionally, the tissue level of these phenolic compounds as well as GSLs can also be modulated by wounding stress alone and in combination with exogenous phytohormones including methyl jasmonate or ethylene.

As a plant architectural trait, branch angle is the basic requirement for high-density cultivation and mechanical harvesting in rapeseed (*Brassica napus*). In a panel of 143 elite *B. napus* accessions, the 60 K Illumina Infinium Single Nucleotide Polymorphisms array analyses revealed significant natural phenotypic variations in branch angle (Liu et al.). Four QTLs for branch angle included two Lazy (AT5G14090) orthologous, SPL14 (AT1G20980) and auxin-responsive GH3 family protein (AT5G51470), genes. Rapeseed possesses significant melliferous potentials and represents a main forage crop for bees. In winter genotypes of field-grown *B. napus* var. oleifera, the volume of the nectar, a nutrient-rich aqueous solution, can vary and occur in the range of 0.02–0.75 μL flower^−1^ (Bertazzini and Forlani). Additionally, the phloem sap composition is modulated by the nectar-variability that, in turn, depends on nectary metabolism.

## Breeding study outcomes

Specific locus amplified fragment sequencing (SLAFS) can be utilized to compare genomic studies within the *B. oleracea* species, QTL identification, and their molecular breeding. In a double-haploid, segregating population of *B. oleracea*, the genetic linkage map constructed with SLAFS revealed a total genetic length of 890.01 cM (with an average marker interval of 0.50 cM), and covered 364.9 Mb of the reference genome (Zhao et al.). Asymmetric somatic hybridization and alien DNA introgression can contribute to the genetic and epigenetic alterations of somatically hybridized black mustard “G1/1” (*B. nigra*, 2*n* = 16, BB genome) introgression lines and can also be a major resource for breeding *B. oleracea* var. *botrytis* (2*n* = 18, CC genome) (Wang et al.). Global transcriptome analyses through RNA-Seq confirmed the expression of transgressive gene in *Brassica* allotetraploids (*B. juncea*, AABB; *B. napus*, AACC; *B. carinata*, BBCC) as genome-wide and temporal, where transgressive up-regulation of resistance-related genes actually controls their immediate physiological pre-adaptation (Zhang et al.). Therein, the silique walls can exhibit much wider variations than leaves after the onset of the tissue-specific expression partitioning quickly after the allotetraploids formation and is associated with the expression of r-protein genes. Notably, the maturity of silique walls and exhibition therein of low translation activity (vs. young leaves) can control the exhibition of expression levels of the r-protein gene in silique lower than in leaves. This can be further corroborated with the expression of the rRNA genes (silenced in vegetative tissues) in reproductive tissues such as sepals and petals. Information is meager on the microRNAs (miRNAs) underlying flower bud development and their potential response to the Ogura-cytoplasmic male sterility (Ogura-CMS), a CMS type with complete male sterility and stability in Chinese cabbage (*B. rapa* ssp. *pekinensis*). *B. rapa* ssp. *pekinensis* buds from both Ogura-CMS and its maintainer possess 426 novel miRNAs, where a regulatory network involving two novel miRNA/target cascades (novel-miR-335/H+-ATPase and novel-miR-448/SUC1) contributes to bud development, especially for pollen engenderation (Wei et al.). Cytogenetic diversity of simple sequence repeats (SSRs) among morphotypes of *B. rapa* ssp. *chinensis* can be revealed by fluorescence *in situ* hybridization. Besides, this method can also confirm nonrandom and motif-dependent chromosomal locations of mono-, di-, and tri-nucleotide repeat loci (Zheng et al.). In fact, differences between SSR repeats with respect to abundance and distribution lead to the genomic evolution of *B. rapa* species. In order to profile the genome-wide DNA methylation, the available methods so far, such as whole-genome bisulfite sequencing (including MethylC-seq and BS-seq), perform single-cytosine methylation resolution and directly estimate the proportion of molecules methylated. Whereas, enrichment of CG-rich sequences in the genome can be possible with the reduced representation bisulfite sequencing (RRBS) method. A new double enzyme-digested RRBS method exhibits a consistent percentage of CG, CHG, and CHH loci located in genic regions between enriched targeted regions and can help in genome-wide DNA methylation profiling at single-base resolution in plants such as *B. rapa* (Chen et al.). The modified RRBS can be a cost-effective, simple, and suitable method for DNA methylation-profiling of large natural populations or for construction of DNA methylation genetic maps.

There occurs a crosstalk between anthocyanin and photosynthesis in *Brassica* species with purple or green leaves. Therein, the upregulation of both transcription factors (TFs), including *Transparent Testa* 8 (*TT8*), and *Transparent Testa 19* (*TT19*), and the anthocyanin late biosynthetic genes (LBGs), especially *dihydroflavonol 4-reductase* (*DFR*) and *anthocyanidin synthase* (*ANS*), contributes to anthocyanin production (Mushtaq et al.). Notably, the upregulation of cytosolic 6-phosphogluconolactonase (*PLG5*) that is involved in the oxidative pentose phosphate pathway points toward the involvement of photosynthesis in the purple color of leaves. Additionally, downregulation of three genes *FTSH PROTEASE 8* (*FTS8*), *GLYCOLATE OXIDASE 1* (*GOX1*), and *GLUTAMINE SYNTHETASE 1;4* (*GLN1;4*) related with the degradation of photo-damaged proteins in photosystem II and light respiration also corroborates the photosynthesis role in the purple color of leaves. In 29 populations of microspore-derived plantlets from cabbage (*B. oleracea* var. *capitata*) and broccoli (*B. oleracea* var. *italica*), the chromosome doubling can be random and genotype-dependent (Yuan et al.). Compared with the result of the total spontaneous doubling of 0 to 76.9% and 52.2 to 100% in 14 *B. oleracea* var. *capitata* and 15 *B. oleracea* var. *italica* populations, respectively, immersing microspore-derived haploid plantlet roots of *B. oleracea* var. *capitata* or *B. oleracea* var. *italica* in colchicine can artificially double chromosomes to over 50%. Formation of adventitious roots (AR) is of great importance for vegetative propagation, but difficult to achieve in many crop species. Aeroponic systems with varying root zone temperatures without using any plant hormones can successfully generate AR from stem-segment explants of *Brassica* species in less than a week. In *B. alboglabra, B. oleracea* var. acephala, *B. rapa* var. nipposinica, and *B. rapa* ssp. chinensis, maintaining cool root zone temperature (C-RZT; 20 ± 2°C) and ambient root zone temperature (A-RZT; 30 ± 2°C) significantly control the water and nutrient uptake capacity and stomatal conductance and differentially contribute to their productivity (Srikanth et al.).

## Stress management and mineral nutrition

Plant salt tolerance involves very complex mechanisms and varies with plant developmental and polyploidy levels. Notably, *Brassica* genotypes differing in ploidy level vary in their tolerance to salinity stress, where microsatellite (SSR) markers can help in their molecular breeding for salt tolerance (Kumar et al.). *Brassica* and *Arabidopsis* share the same family Brassicaceae, wherein an SSR marker-assisted comparative molecular marker mapping can contribute to the development of robust and healthy plants with high yield potential. *AtDjA3* gene encoding a heat shock protein 40 (J-protein) can be modulated by salt and osmotic stress. Notably, exhibition of differential seed morphology and sensitivity to salt, glucose, and ABA in *Atdja3*-null mutant line (j3) involves high transcript levels of ABA-insensitive 3 (Salas-Muñoz et al.). Among the ten classes of glutathione transferases (GSTs), known for their diversified important roles in plants, the phi, tau, lambda, and DHAR classes of GSTs are considered unique to plants. *Capsella rubella*, a member of the mustard family and a close relative of *A. thaliana*, possesses 49 GST genes coming within eight classes (He et al.). Additionally, *Capsella* and *Arabidopsis* GSTs exhibit functional divergence (both in gene expression and enzymatic properties) in paralogous gene pairs in *Capsella* (even the most recent duplicates), and orthologous GSTs in *Arabidopsis*/*Capsella*.

The nitrogen-efficiency capacity of plants such as *B. juncea* is modulated by N-supply and elevated [CO_2_] conditions, where proteins like *PII*-like protein, cyclophilin, elongation factor-TU, oxygen-evolving enhancer protein, and rubisco activase are involved in maintaining photosynthesis, energy metabolism, and overall plant health (Yousuf et al.). Though regarded earlier as an environmental pollutant and a biotoxic agent, H_2_S can play multiple functions in plants and improve cellular pools of nutrients including Ca, Cu, Fe, Mg, Mn, Na, and P under deficiency of certain element such as S (Reich et al.). For instance, in *Brassica pekinensis* (Lour.) Rupr. cv. Kasumi F1, H_2_S partially prevented S-deficiency caused increase in the levels of Mo (and also Zn) that was argued a result of partial down-regulation of the sulfate transporters. Among important Ca^2+^ sensor proteins, calmodulins (CaMs) bind to certain TFs such as calmodulin-binding transcription activators (CAMTAs) and play important roles in various plant disease resistances and abiotic stress tolerances. Notably, *B. napus*, a tetraploid of the two progenitors *B. rapa* and *B. oleracea*, possess 18 CAMTAs, the highest number of CAMTAs among over 40 plant species studied for the same, and also 3-folds as many as that in *Arabidopsis* (Rahman et al.). *Arabidopsis* CAMTA mutant (AtCAMTA3) negatively regulates the resistance to *Sclerotinia sclerotiorum*, an ascomycete necrotrophic fungus, which induces the expression of *BnCAMTA3A1* and *BnCAMTA3C1*.

## Environmental perspectives

In addition to its role in plant growth, the separated (monoculture) and combined (simultaneous) cropping of plants controls important processes at rhizosphere, solubility/bioavailability and accumulation/remediation of contaminants. Co-cropping of an *Alyssum murale* Ni-hyperaccumulator ecotype with nonhyperaccumulator *A. montanum* and perennial ryegrass (*Lolium perenne*) has no affect on Ni accumulation in *A. murale* leaves and stems (Broadhurst and Chaney). However, co-cropping of *L. perenne* with *A. murale* can help the former to accumulate Cu up to 10 mg kg^−1^, independent of the phytosiderophores. Additionally, Mn mobilization by the *Alyssum* hyperaccumulator species can significantly increase Mn levels in *L. perenne*. *Sinapis alba*, known as yellow/white mustard, is an important cruciferous condiment crop and also contributes to environmental pollutants-cleanup. In *S. alba* leaves, stems, and roots by *de novo* transcriptome analysis revealed the expression of 3,489, 1,361, and 8,482 unigenes, respectively (Zhang et al.). Genes pre-dominantly expressed in the leaves were enriched in photosynthesis- and C fixation-related pathways; whereas, stem-dominant genes were related with pathways related to sugar, ether lipid, and amino acid metabolisms and plant hormone signal transduction and circadian rhythm pathways. On the contrary, the root dominant genes were enriched in pathways related to lignin and cellulose syntheses involved in plant pathogen interactions, and potentially responsible for heavy metal chelating and detoxification. Though the oxidation of Glutathione (GSH) into GSSG occurs in leaves, its extensive conversion into PCs takes place in roots. Clues can be useful in the research and molecular breeding of *S. alba* and also for the molecular-assisted transfer of beneficial traits to other crops.

## Conclusions and outlook

Most of the contributions discussed molecular-genetic insights into agri-horticultural aspects that can help in devising breeding approaches in the members of the Brassicaceae family. Stress management and nutrition in *Brassica*, the second most discussed aspect, had little discussion on the environmental perspectives. Overall, this research topic yielded notable results/observations related to the yield and nutritional quality of and insights important for breeding *Brassica*. Studies on QTLs for branch angle, quantitative trait PDgr, and rRNA genes can significantly improve photosynthesis efficiency, biomass, flower and silique, and overall yield in *Brassica*. Studies are imperative for unveiling molecular insights into low N supply and elevated [CO_2_] influences on the major plant proteins/enzymes and H_2_S-mediated minimization of deficient-S accrued impacts in *Brassica*. The co-cropping of nonhyperaccumulator and metal-hyperaccumulator ecotypes has yielded promising results; however, more exhaustive studies are required in this direction to get benefit of *Brassica* in the environmental perspective. It is suggested, herein, to conduct more intensive research on breeding crops within the Brassicaceae family for improved health, productivity, and quality. In addition, more insights into the contribution of Brassicaceae family members to environmental issues also need to be addressed in future studies on the subject.

## Author contributions

NA and SG prepared the first draft of the manuscript. OD, JJ, and NT read and revised the manuscript, and all authors listed approved the final version for publication.

### Conflict of interest statement

The authors declare that the research was conducted in the absence of any commercial or financial relationships that could be construed as a potential conflict of interest.

